# Induced Hydroxylation on Exfoliated Boron Nitride: Photocatalytic and Adsorptive Properties

**DOI:** 10.3390/molecules31101616

**Published:** 2026-05-11

**Authors:** María Mónica Hernández-Orozco, Fabiola Hernández-Rosas, Rusbel Eduardo Trinidad-Urbina, Rafael Ramírez-Bon

**Affiliations:** 1 Centro de Investigación y de Estudios Avanzados del IPN, Unidad Querétaro, Apdo. Postal 1-798, Querétaro 76001, Mexico; monica.hernandezo@cinvestav.mx (M.M.H.-O.); rusbel.trinidad@cinvestav.mx (R.E.T.-U.); 2 Escuela de Ingeniería Biomédica, División de Ingeniería, Universidad Anáhuac Querétaro, Cto. Universidades I, Fracción 2, Querétaro 76246, Mexico

**Keywords:** hydroxylated boron nitride, dye removal, ROS generation

## Abstract

Hexagonal boron nitride (h-BN) is a chemically stable two-dimensional material whose wide band gap and low surface reactivity limit its performance in adsorption and photocatalysis, motivating strategies to tailor its structure. In this work, a mechanochemical approach combining high-energy ball milling with NaOH-assisted treatment was used to induce simultaneous exfoliation and hydroxylation of h-BN, promoting defect generation, reduced crystallinity, interlayer expansion, and incorporation of oxygen-containing groups (B-OH and B-O). These modifications led to band gap narrowing, increased surface polarity, and improved dispersion, enabling the formation of heterogeneous active sites. The hydroxylated material (BN-OH) exhibited high adsorption capacities of 248 mg/g for methylene blue (MB) and 215 mg/g for rhodamine 6G (R6G), following Freundlich behavior, indicative of heterogeneous adsorption governed by electrostatic interactions, π–π stacking, hydrogen bonding, and defect-mediated sites. Under solar irradiation, BN-OH achieved up to 99% degradation of both dyes, following predominantly pseudo-first-order kinetics and outperforming pristine BN; additionally, the kinetic behavior under solar conditions was successfully described using the Behnajady–Modirshahla–Ghanbary (BMG) model, which accurately predicts the two-stage degradation process. Scavenger experiments revealed that ⦁OH radicals dominate MB degradation, while ⦁OH, O_2_⦁^−^, and h^+^ contribute to R6G removal. Overall, defect engineering and hydroxyl functionalization synergistically enhance photocatalytic performance, providing a scalable strategy for wastewater treatment.

## 1. Introduction

In recent years, hexagonal boron nitride (h-BN) has attracted increasing attention as an emerging two-dimensional material due to its layered structure analogous to graphene, which is why it is also referred to as “white graphene” [1]. This material is composed of hexagonal layers of boron and nitrogen atoms bonded by strong covalent interactions, with adjacent layers held together by van der Waals forces [2]. This unique structure provides h-BN with remarkable properties such as high chemical and thermal stability, excellent mechanical strength, high thermal conductivity, and outstanding structural stability, making it a promising candidate for applications in catalysis, electronics, energy storage, and materials science [3]. Despite these advantages, bulk h-BN has a wide bandgap (5.5–6 eV), which makes it an electrical insulator and limits its activity in photoinduced processes [3,4]. However, recent studies have shown that its properties can be tailored through strategies such as exfoliation, the introduction of structural defects, or surface functionalization, enabling the tuning of its electronic structure and enhancement of its surface reactivity [5,6,7]. On the other hand, mechanochemical approaches such as high-energy ball milling have emerged as efficient strategies for the structural modification of layered materials like h-BN. Unlike conventional chemical methods, which are typically limited to surface functionalization, ball milling induces defects directly within the crystal lattice, including dislocations, reduced crystallite size, and increased structural disorder [8]. This process also promotes partial exfoliation of the layers, leading to an increase in surface area and the density of active sites, ultimately enhancing surface reactivity. For instance, Fu et al. reported the use of ball-milled h-BN as an active component in ZnO-based photocatalytic systems, where the mechanically treated material acted as a hole-transfer promoter and as a site for electrostatic interaction with charged dye molecules. The authors demonstrated that the milling process not only modifies the surface charge of h-BN, but also facilitates the separation of photogenerated charge carriers, significantly improving the photocatalytic efficiency of the ZnO/h-BN system in the degradation of cationic dyes [9].

Regarding the environmental field, semiconductor solid materials have gained significant attention as catalysts in advanced oxidation processes (AOPs), particularly in heterogeneous photocatalytic systems. These processes rely on the in situ generation of highly reactive species, such as hydroxyl radicals (•OH) and other reactive oxygen species, which can oxidize persistent organic compounds [10]. The performance of these systems strongly depends on the structural, optical, and surface properties of the catalyst, as well as on the operating conditions, such as pH. For this reason, the surface modification of semiconductor materials through the introduction of structural defects and functional groups has become an effective strategy to enhance the generation of reactive species and the separation of photogenerated electron–hole pairs [11]. The development of efficient materials for these processes is particularly relevant to attend to the growing problem of water contamination by industrial effluents, especially those originating from the textile, pharmaceutical, cosmetic, and food industries, which discharge large amounts of organic pollutants into aquatic environments [12]. Among these, some industrial dyes such as methylene blue (MB) and rhodamine 6G (R6G) exhibit high chemical stability and resistance to degradation, allowing them to persist in aquatic ecosystems and negatively affect photosynthetic processes in aquatic organisms [13].

In this context, surface-modified h-BN has emerged as a promising alternative for environmental applications [14]. The hydroxylation of boron nitride enables the incorporation of functional groups such as –OH, as well as B–O and N–O bonds on its surface, generating structural defects that increase surface polarity [15,16]. These defects enhance both adsorption processes and photocatalytic activation through the formation of reactive oxygen species. For instance, Al Noman et al. [17] reported the hydroxylation of h-BN sheets using a chemical weathering method and evaluated their ability to remove anionic dyes, such as Congo Red (CR), and cationic dyes such as Neutral Red (NR) from aqueous solutions at different pH values. The authors reported removal efficiencies of 99.23% for CR at pH 2 and 85.77% for NR at pH 11. Similarly, Ji et al. [18] synthesized BN via a calcination method and observed that the presence of hydroxyl groups in its structure reduces the band gap from approximately 5.5 eV to 3.94 eV, enabling the photocatalytic degradation of dyes such as rhodamine B, methylene blue, and methyl orange, with efficiencies of 45.2%, 12.2%, and 7.4%, respectively. Despite these advances, the available information regarding the application of hydroxylated boron nitride in water treatment remains limited, particularly regarding its combined performance in adsorption and photocatalysis processes.

In this work we report the hydroxylation of boron nitride by means of a NaOH-assisted high-energy ball milling process and evaluated its adsorptive and photocatalytic performance using methylene blue and rhodamine 6G as model dyes. The material modifications were examined through structural, morphological, surface, and optical characterization. The adsorption behavior of the hydrolyzed BN material is analyzed using Freundlich, Langmuir, and Temkin isotherm models to elucidate the interaction mechanisms between the material and the contaminants. Furthermore, its photocatalytic activity is evaluated through photodegradation kinetic studies, fitting the experimental data to pseudo-first-order and pseudo-second-order models. This study aims to contribute to the development of advanced, sustainable materials based on modified h-BN for potential application in the treatment of contaminated effluents.

## 2. Results

### 2.1. Morphological and Compositional Characterization

**Figure 1:**
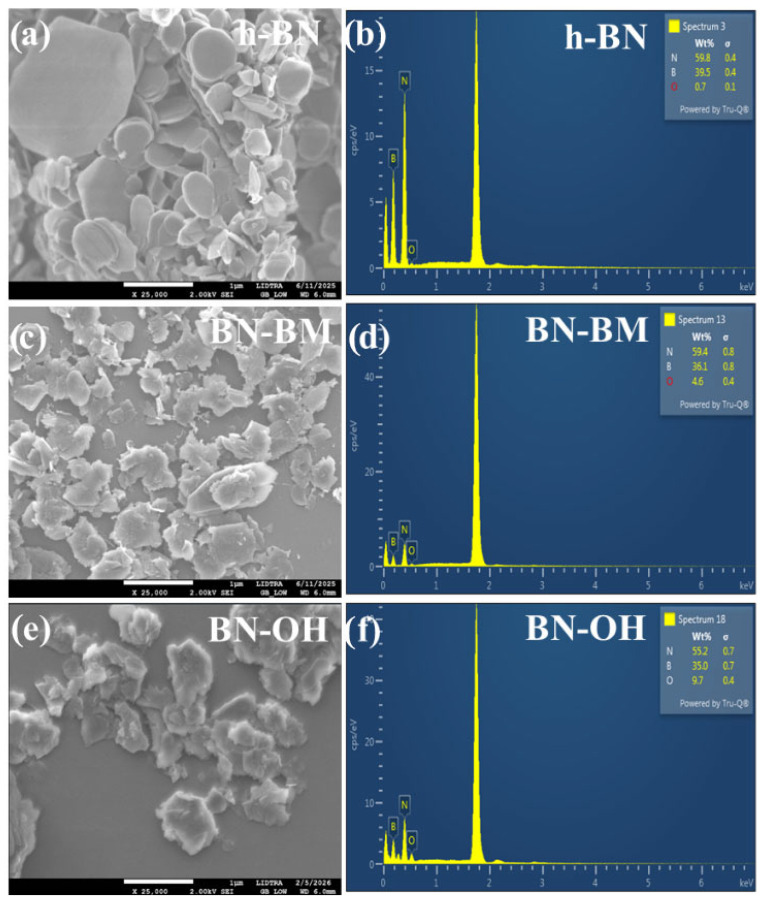
SEM micrographs and corresponding EDS spectra of (**a**,**b**) pristine h-BN, (**c**,**d**) ball-milled BN (BN-BM), and (**e**,**f**) hydroxylated BN (BN-OH).

The morphological and compositional characterization of boron nitride was carried out to evaluate the structural changes induced by the mechanical and chemical treatments applied to the material, as shown in Figure 1. Three powder systems were analyzed: untreated BN (denoted as BN), BN subjected to dry mechanical ball milling for 3 h (denoted as BN-BM), and BN treated by milling in the presence of a 2 M NaOH solution (denoted as BN-OH). The SEM micrograph in Figure 1a reveals that untreated boron nitride exhibits the typical platelet-like morphology associated with hexagonal BN, characterized by stacked lamellar structures with relatively smooth surfaces [19]. After dry mechanical milling for 3 h, as shown in Figure 1c, the BN platelets undergo significant fragmentation, producing smaller flakes with irregular edges, indicating partial mechanical exfoliation of the layered structure [20]. Figure 1d shows that when the BN milled material is subsequently treated with a 2 M NaOH solution under additional milling, its morphology becomes more irregular and aggregated, suggesting chemical attack and structural modification of the BN layers [21]. EDS analysis revealed the characteristic elemental composition of boron nitride under the different treatment conditions. For pristine BN (Figure 1b), weight percentages of 39.5% boron (B), 59.8% nitrogen (N), and 0.7% oxygen (O) were obtained. In the case of BN-BM (Figure 1d), the composition was 36.1% B, 59.4% N, and 4.6% O, whereas BN-OH exhibited 35.0% B, 55.2% N, and 9.7% O (Figure 1f). There is an increase in oxygen indicating the incorporation of oxygen-containing functional groups, likely associated with surface hydroxylation and the formation of structural defects in the BN framework [22]. The red color in the EDS tables is used only to identify the oxygen contribution detected by EDS and does not correspond to spatial elemental mapping or morphological contrast.

### 2.2. Structural and Surface Chemical Characterization

**Figure 2:**
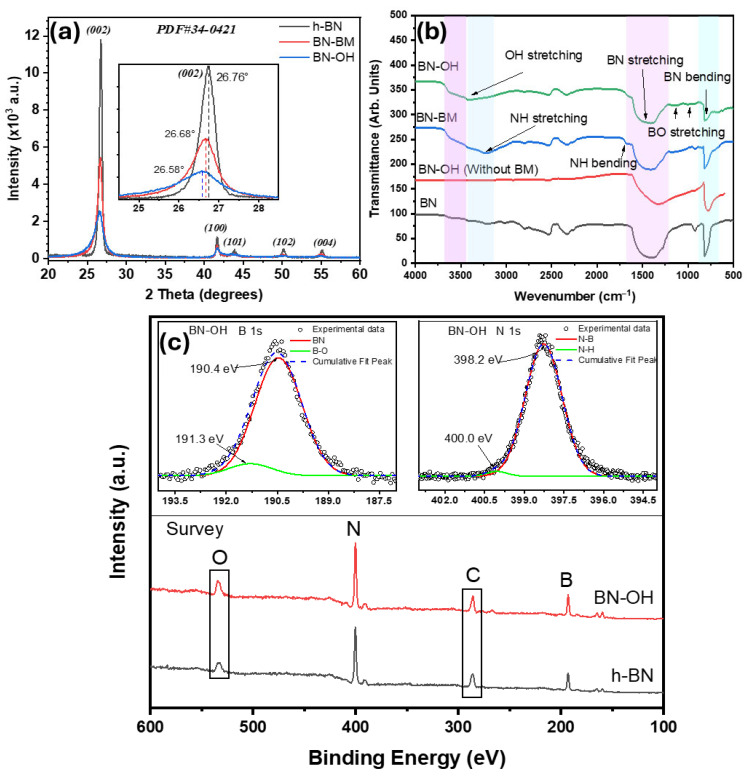
Structural, vibrational, and surface chemical characterization of boron nitride samples: (**a**) XRD patterns, (**b**) FTIR spectra, and (**c**) high-resolution XPS spectra of B 1s and N 1s for pristine BN and BN-OH. The reference pattern PDF#34-0421 corresponds to hexagonal boron nitride (h-BN).

These results are further supported by the X-ray diffraction (XRD) patterns shown in Figure 2a. The diffraction patterns exhibit the characteristic peaks of hexagonal boron nitride (h-BN), confirming the presence of the typical laminar structure of this material. The observed peaks are consistent with the hexagonal boron nitride phase, with no detectable secondary phases within the sensitivity limit of the technique. The main peak associated with the (002) plane is observed at approximately 26–27°, which agrees with reported values for h-BN (PDF No. 34-0421). Pristine BN exhibits the (002) peak at 26.75°, with an interplanar spacing (d_002_) of 0.332 nm, a full width at half maximum (FWHM) of 0.402°, and an estimated crystallite size of 20.3 nm. After ball milling (BN-BM), the (002) peak slightly shifts to 26.68°, accompanied by an increase in d_002_ to 0.333 nm, an increase in FWHM to 0.61°, and a reduction in crystallite size to 13.3 nm. In the case of BN-OH, the (002) peak is located at 26.58°, with a d_002_ of 0.335 nm, an FWHM of 0.75°, and a crystallite size of 10.8 nm. These changes, together with the decrease in crystallinity from 80.92% to 53.49% and 39.13% for BN, BN-BM, and BN-OH, respectively, suggest that the mechanical and chemical treatments induce an increase in structural disorder within the material [8]. The structural parameters obtained from XRD analysis are summarized in Table 1. The shift of the (002) peak toward lower 2θ values indicates a slight increase in interlayer spacing, which can be associated with the generation of structural defects and the possible incorporation of surface functional groups during the hydroxylation process. Consistently, the progressive increase in FWHM indicates a reduction in crystallite domain size and a decrease in long-range crystalline order [23].

The surface functional groups of BN, BN-BM, and BN-OH were analyzed by Fourier transform infrared (FTIR) spectroscopy. In all samples, their FTIR spectra, shown in Figure 2b, display the characteristic bands of hexagonal boron nitride corresponding to the B–N stretching vibration in the 1360–1400 cm^−1^ region and the out-of-plane B–N–B vibration between 780 and 820 cm^−1^ [24,25], confirming the preservation of the basal BN structure after the applied treatments. In the modified materials, changes are observed in the 1000–1200 cm^−1^ region, associated with B–O vibrations, suggesting oxygen incorporation and the formation of surface species such as B–OH or B–O^−^, particularly in the NaOH-treated sample [22,26]. Additionally, bands are detected in the 3300–3500 cm^−1^ and 1500–1650 cm^−1^ regions, attributed to N–H vibrations, which are related to the exposure of nitrogen edge sites and the generation of defects or terminal groups induced by exfoliation and chemical treatment [20]. The increased intensity of these signals in both BN-BM and BN-OH supports that the treatments promote chemical activation of the boron nitride edges. For comparison, a control hydroxylation without ball milling was also performed. In this case, the FTIR spectrum of pristine BN, subjected to ultrasonic treatment (1 h) followed by NaOH stirring at 60 °C for 3 h, shows only minor spectral changes compared to BN. The absence of significant new bands or intensity enhancement in the B–O (~1000–1200 cm^−1^) and O–H (~3200–3500 cm^−1^) regions suggests that hydroxylation is limited under these conditions.

High-resolution XPS spectra of B, N, C, and O confirm the characteristic chemical composition of hexagonal boron nitride, as shown in Figure 2c. In the survey spectrum, the BN-OH material exhibits a higher relative contribution of the O 1s signal and, to a lesser extent, C 1s as compared to pristine h-BN, indicating surface enrichment in oxygen-containing species after alkaline treatment. The increase in the O 1s signal is consistent with the incorporation of oxygenated functional groups. The B 1s (190.4–190.6 eV) and N 1s (398.2–398.5 eV) signals correspond to B–N bonds in the crystalline lattice, in agreement with reported values for h-BN [17]. In the BN-BM sample, the B–N structure is preserved, as evidenced by the main B 1s peak (190.5 eV); however, an additional contribution appears in the N 1s region around 399 eV, attributed to nitrogen species associated with structural defects induced by ball milling [27]. In contrast, BN-OH retains the base structure of the material but exhibits a secondary contribution in the B 1s region at higher binding energies (191.3 eV), which is associated with the formation of B–O and/or B–OH bonds, indicating partial surface functionalization. Likewise, in the N 1s region, a secondary component (399.5–400.0 eV) is observed, related to defect sites or perturbed chemical environments, likely arising from interactions with hydroxyl groups and the generation of active surface sites [28]. These contributions are clearly observed in the inset images, where the high-resolution deconvolutions of the B 1s and N 1s regions for BN-OH are presented, evidencing the coexistence of B–N bonds with functionalized and defect-related species.

### 2.3. Physicochemical and Optical Properties

**Figure 3:**
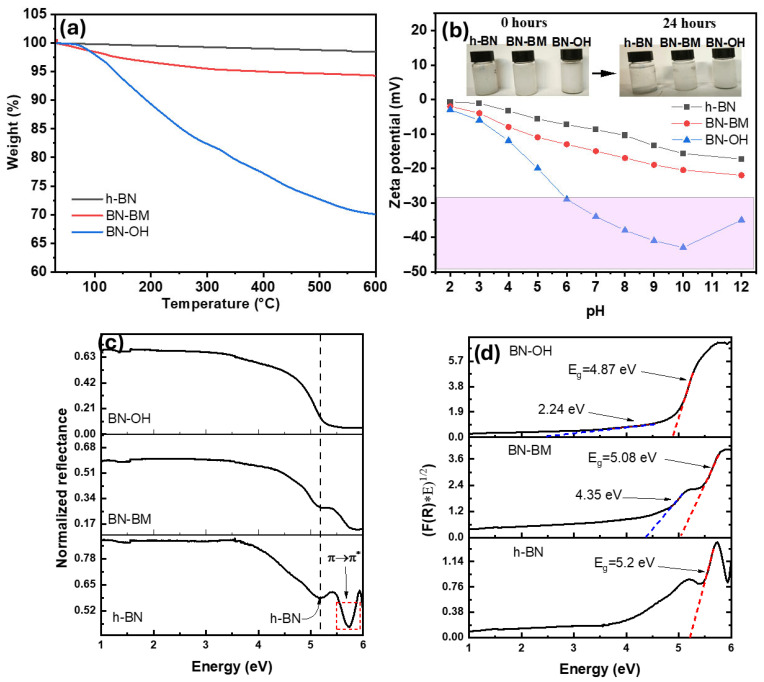
(**a**) Thermogravimetric analysis (TGA), (**b**) zeta potential measurements, (**c**) normalized diffuse reflectance spectra and (**d**) optical band gap determination of BN, BN-BM, and BN-OH.

On the other hand, the TGA curves in Figure 3a show a progressive increase in weight loss after exfoliation and, more markedly, after NaOH treatment. Pristine BN retains a residual mass of approximately 98–99% at 600 °C, reflecting its high thermal stability and low content of volatile surface species [29,30]. In contrast, the modified materials BN-BM and BN-OH exhibit significantly higher weight losses, approximately 5% and 30% higher, respectively, compared to pristine BN. The higher weight loss observed in BN-BM and BN-OH is primarily attributed to the introduction of structural defects and oxygen-containing functional groups induced by mechanochemical treatment, which, due to their thermal instability, decompose upon heating, resulting in the observed mass loss [31]. High-energy ball milling promotes exfoliation and defect formation, which facilitates the incorporation of hydroxyl and B–O species [22], as confirmed by FTIR and XPS analyses. Surface area modification is expected due to exfoliation [20]; it primarily contributes by providing additional active sites for functionalization rather than directly causing weight loss. These results confirm that the degree of surface functionalization follows the order BN-OH > BN-BM > BN, while thermal stability decreases in the same trend.

In addition, the surface charge of the materials was evaluated from the zeta potential measurements shown in Figure 3b. The shaded background in Figure 3b highlights the pH range in which BN-OH exhibits a more negative zeta potential compared with pristine BN and BN-BM. Pristine BN exhibits negative zeta potential values over the entire pH range, varying from −0.7 to −17.5 mV, indicating a low density of ionizable groups and an electrostatically stable surface [32]. In comparison, BN-BM shows more negative zeta potential values across the full pH range, which is attributed to the increase in surface area and the generation of defects and active sites, promoting the deprotonation of surface groups. In contrast, the hydroxylated material (BN-OH) displays a sharp decrease in zeta potential under neutral and alkaline conditions, because of the incorporation of hydroxyl groups during the alkaline treatment and their subsequent deprotonation [33]. The increase in the anionic character and electrostatic stability of the BN materials induced by ball milling and hydroxylation treatments enhances their performance in adsorption and photocatalytic applications. These results are consistent with the dispersion tests in deionized water, shown in the inset of Figure 3b, where suspensions prepared with 10 mg of material in 5 mL of water and subjected to sonication exhibited different sedimentation behaviors over time. While pristine BN rapidly settled, forming a clear supernatant, BN-BM showed a slightly more stable dispersion. In contrast, BN-OH remained visibly dispersed even after prolonged resting periods, indicating a higher affinity for the aqueous medium.

Finally, the normalized diffuse reflectance spectra (DRS) of BN, BN-BM, and BN-OH are presented in Figure 3c, revealing noticeable changes in the electronic transitions after the mechanochemical treatments. To better analyze these optical modifications, the spectra were transformed using the Kubelka–Munk function [34] and the Tauc method to estimate the band gap values, assuming allowed indirect transitions, characteristic of hexagonal boron nitride (h-BN). As shown in Figure 3d, pristine boron nitride exhibited a band gap of approximately 5.2 eV, which falls within the range reported for commercial h-BN powders [35,36]. After mechanical exfoliation (BN-BM), the band gap slightly decreased to 5.08 eV. Additionally, an extra contribution around 4.35 eV is observed in the spectrum. In the case of the hydroxylated material (BN-OH), a more pronounced reduction in the band gap to 4.87 eV was observed, along with an additional feature around 2.24 eV. The observed evolution of the optical absorption features in BN-BM and BN-OH is consistent with previous reports on functionalized boron nitride systems. Liu et al. demonstrated, through combined experimental and DFT analysis, that the incorporation of hydroxyl and amino groups in porous h-BN can modify its electronic structure and lead to a reduction in the optical band gap, accompanied by an extension of light absorption toward lower energies [37]. In parallel, Angizi et al. [38] reported that surface and edge functionalization of BN nanostructures induces the formation of additional electronic states within the band gap, resulting in multiple optical transitions rather than a single well-defined absorption edge. These defect-related states arise from orbital hybridization, charge transfer effects, and structural disorder introduced during functionalization. Therefore, these results demonstrate the modulation of the optical and electronic properties of these materials, extending their absorption toward lower energy regions. This band gap modification may enhance electronic interactions with adsorbed species and improve the performance of the material in adsorption and photocatalytic applications.

### 2.4. Adsorption Performance and Isotherm Analysis

**Figure 4:**
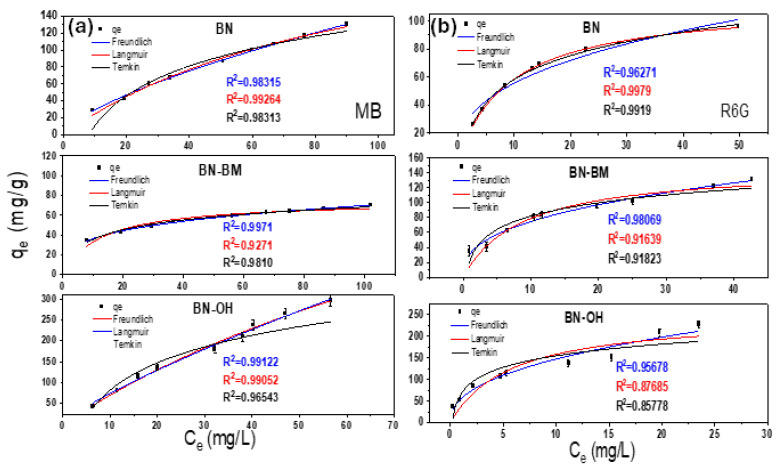
Adsorption isotherms of (**a**) MB and (**b**) R6G on BN, BN-BM, and BN-OH fitted with Langmuir, Freundlich, and Temkin models. The black square symbols represent the experimental adsorption capacity (q_e_), while the blue, red, and black lines correspond to the Freundlich, Langmuir, and Temkin fitting models, respectively. The R^2^ values are shown using the same color code as the corresponding fitting curves.

The adsorption isotherms of MB and R6G for BN, BN-BM, and BN-OH were obtained by plotting the amount of adsorbate (dyes) per adsorber (BN) mass, qe (mg/g), versus the concentration of the adsorbate remaining in equilibrium in solution at a constant temperature, Ce (mg/L). Figure 4 shows the experimental results and the fittings to the Langmuir, Freundlich, and Temkin models, while the corresponding isotherm parameters are summarized in Table 2. For both dyes, the adsorption capacity follows the same trend among the materials, with BN-OH exhibiting the highest performance. Notably, MB shows higher adsorption capacities (up to 248 mg/g for BN-OH) compared to R6G (up to 215 mg/g). The Langmuir model adequately describes the behavior of pristine BN (R^2^ = 0.99), indicating monolayer adsorption on relatively homogeneous sites [39]. In contrast, the Freundlich model provides the best fit for BN-BM and BN-OH for both MB and R6G (R^2^ ≈ 0.95–0.99), suggesting adsorption on heterogeneous surfaces with a non-uniform distribution of active sites and energies [40]. The Temkin model also shows good agreement for all systems (R^2^ = 0.85–0.96), indicating that the heat of adsorption decreases linearly with increasing surface coverage, consistent with adsorbate–adsorbent interactions [41]. The difference in adsorption behavior between the dyes is primarily attributed to their molecular structure. MB, with a smaller and more planar configuration, can more easily diffuse toward active sites and establish stronger π–π interactions with the BN surface. In contrast, the bulkier structure of R6G induces steric hindrance, limiting its accessibility to pores and defect sites [42,43]. While steric hindrance plays a role, it is not the sole determining factor. MB exhibits a more compact and planar molecular structure, which facilitates its diffusion toward active sites and enhances its interaction with the structural defects and surface hydroxyl groups in BN-OH. In contrast, R6G possesses a bulkier xanthene-based structure, which limits its accessibility to active sites, particularly in heterogeneous and defect-rich surfaces [42,43,44]. Overall, the adsorption process is governed by a synergistic mechanism involving electrostatic interactions, π–π stacking, hydrogen bonding, and adsorption at defect-induced active sites.

### 2.5. Photocatalytic Activity Under Dark and Solar Conditions

**Figure 5:**
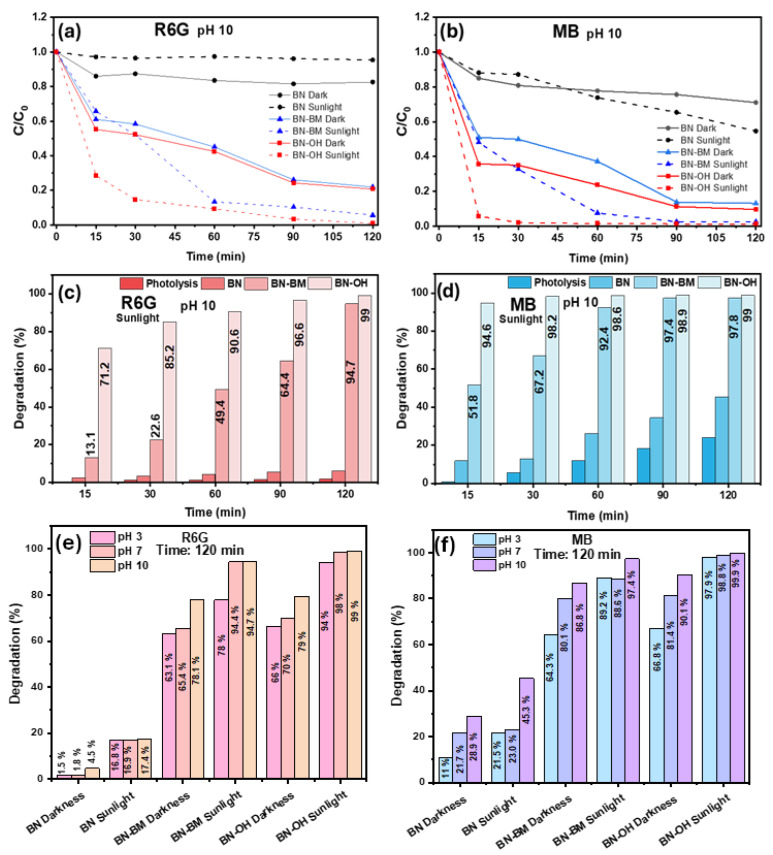
Photocatalytic degradation of (**a**) R6G and (**b**) MB expressed as C/C_0_ versus time, and corresponding degradation efficiencies for (**c**) R6G and (**d**) MB over BN, BN-BM, and BN-OH under dark and sunlight conditions, (**e**) R6G and (**f**) degradation efficiency after 120 min under dark and sunlight conditions at different pH values.

The normalized concentration curves (C/C_0_), shown in Figure 5 for (a) R6G and (b) MB, display a rapid decrease in C/C_0_ at early times (15 min), especially for the BN modified materials. This behavior is directly reflected in the degradation efficiencies obtained under irradiation as shown in Figure 5 for (c) R6G and (d) MB. For R6G, BN-OH reaches approximately 71.2% degradation at 15 min, followed by BN-BM with 13.1%, while pristine BN shows minimal removal. In the case of MB, this trend is even more pronounced, with BN-OH reaching approximately 94.6% degradation, while BN-BM achieves around 51.8%, indicating higher affinity and reactivity toward this dye. The C/C_0_ curves also allow comparison between dark and irradiated conditions. Under dark conditions (Figure 5a,b, solid lines), the decrease in concentration is gradual and limited, indicating that adsorption is the dominant process. In contrast, under sunlight irradiation (Figure 5a,b, dashed lines), a much more pronounced reduction in C/C_0_ is observed, evidencing the photocatalytic activation of the materials. It is important to note that the degradation efficiencies reported (Figure 5c,d) correspond exclusively to experiments conducted under irradiation and therefore represent the overall removal process, including both initial adsorption and subsequent photocatalytic degradation. For R6G, BN-OH reaches values close to 99% at 120 min, while BN-BM achieves approximately 94.7%, and pristine BN exhibits significantly lower efficiency. For MB, the effect is even more remarkable, with BN-OH achieving nearly complete degradation (99%), followed by BN-BM with 97.8%, whereas BN reaches approximately 45.3%. Overall, the difference between dark and irradiated profiles indicates that the photocatalytic contribution is predominant, especially in the modified materials. Finally, MB exhibits higher degradation percentages compared to R6G across all systems, suggesting greater susceptibility to photooxidative processes, likely due to its molecular structure, charge, and more favorable interaction with the modified material surface [45]. Figure 5e,f show the degradation of R6G and MB at pH 3, 7, and 10 after 120 min. At pH 3, the lowest efficiencies are obtained, with typical values below 20% for BN and around 45–65% for BN-BM and BN-OH, due to surface protonation, which hinders interaction with the dyes. At pH 7, degradation improves significantly, reaching approximately 70–85% for BN-BM and up to 90–95% for BN-OH, indicating more favorable conditions for adsorption and photocatalysis. At pH 10, the highest efficiencies are achieved, particularly for BN-OH, due to surface deprotonation, which enhances electrostatic attraction and photocatalytic activity [46]. From a photocatalytic standpoint, the enhanced activity observed for the modified materials can be primarily attributed to defect-induced electronic states, which play a dominant role in governing the photocatalytic response [47]. As previously mentioned, the structural disorder introduced by ball milling and subsequent hydroxylation likely generates sub-bandgap states that facilitate light absorption under solar irradiation and improve charge carrier separation, thus increasing the generation of reactive oxygen species. In addition, the incorporation of –OH groups enhances surface polarity, promoting stronger electrostatic interactions with cationic dyes and improving interfacial charge transfer processes, which further contributes to degradation efficiency [17].

The following Table 3 summarizes the performance of various reported photocatalysts for the degradation of organic dyes, providing a comparative framework to evaluate the efficiency of the materials developed in this work under similar or representative conditions. As shown, BN–OH exhibits competitive, and in some cases superior performance, achieving up to 98.6% degradation of MB under solar irradiation in short reaction times, comparable to or exceeding several systems operating under visible or UV light [48,49,50,51,52,53,54,55,56]. In addition, compared to other BN-based materials [18,55], the present system demonstrates greater versatility by enabling the degradation of multiple dyes under mild conditions. The novelty of this work lies in the implementation of a simple, scalable mechanochemical-assisted hydroxylation strategy that simultaneously introduces defects and –OH groups, enhancing both adsorption and photocatalytic activity without requiring complex heterojunctions or multi-step synthesis [55,56].

**Figure 6:**
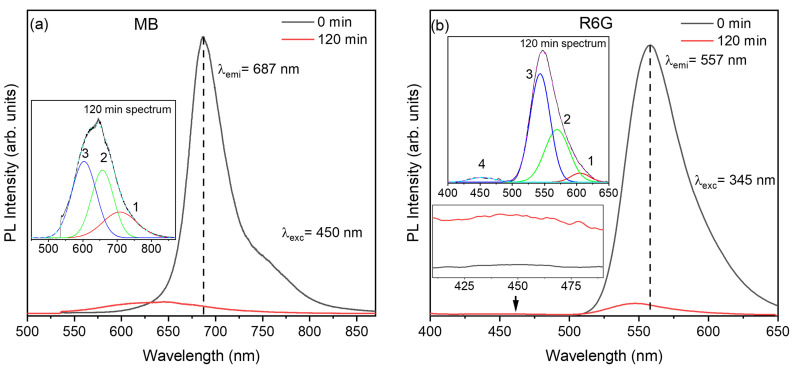
Photoluminescence (PL) spectra of BN–OH after 120 min of solar irradiation during the degradation of (**a**) methylene blue (MB) and (**b**) rhodamine 6G (R6G), including deconvolution of the emission bands. The black and red curves correspond to the PL spectra recorded at 0 and 120 min, respectively. The dashed vertical lines indicate the main emission wavelengths, λem = 687 nm for MB and λem = 557 nm for R6G, while the arrows indicate the excitation wavelengths, λexc = 450 nm for MB and λexc = 345 nm for R6G. The colored curves in the insets represent the deconvoluted emission components of the 120 min spectra.

Furthermore, photoluminescence (PL) measurements were performed at 0 and 120 min of solar irradiation to gain insight into the formation of intermediate species during the degradation process. As shown in Figure 6, the emission spectra of both dyes exhibit a significant decrease in intensity after irradiation, accompanied by spectral broadening and the emergence of additional features, indicating the formation of new fluorescent species. For methylene blue (Figure 6a), the deconvolution of the 120 min spectrum reveals three main components: a band centered around ~680–690 nm associated with the residual monomeric dye, a second contribution at ~610–630 nm attributed to partially demethylated intermediates (e.g., azure-type species), and a third band at lower wavelengths (~580–600 nm), which can be related to further degradation products and/or smaller conjugated fragments [57,58]. In the case of rhodamine 6G, the deconvoluted spectrum at 120 min shows multiple contributions, including a dominant band near ~550–560 nm corresponding to the remaining monomer, along with additional components at ~520–540 nm and ~580–600 nm, which are associated with intermediate species such as deethylated derivatives and aggregated forms arising during the degradation process [59,60]. These spectral changes provide clear evidence that the observed dye removal is not limited to simple decolorization, but involves progressive chemical transformation of the molecules.

### 2.6. Kinetic Analysis

**Figure 7:**
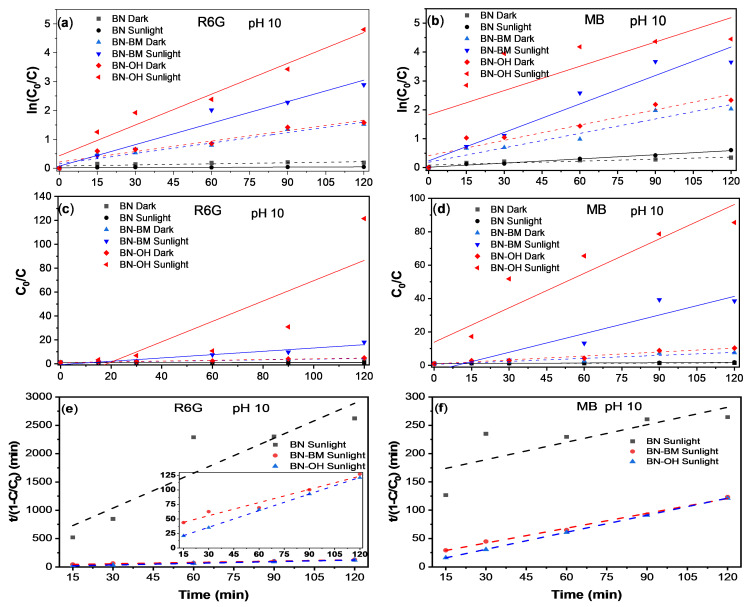
Kinetic analysis of R6G and MB degradation using pseudo-first-order (PFO), pseudo-second-order (PSO), and Behnajady–Modirshahla–Ghanbary (BMG) models for BN, BN-BM, and BN-OH under dark and sunlight conditions at pH 10. (**a**,**b**) PFO kinetic fitting plots for R6G and MB degradation, respectively; (**c**,**d**) PSO-related C_0_/C profiles for R6G and MB degradation, respectively; and (**e**,**f**) BMG linear fitting plots for R6G and MB degradation under solar irradiation, respectively. The colored symbols identify the different BN-based samples and experimental conditions, while the corresponding lines represent the fitting curves. The dotted lines in panels (**e**,**f**) correspond to the BMG linear fittings.

The degradation kinetics of the dyes were evaluated at pH 10, the previously identified optimal condition, and the results are presented in Figure 7a–d. The linearized plots corresponding to the pseudo-first-order (PFO) and pseudo-second-order (PSO) models allow analysis of the kinetic behavior of BN, BN-BM, and BN-OH systems under dark and irradiation conditions. In the PFO plots (ln(C_0_/C) vs. time), shown in Figure 7 for (a) R6G and (b) MB, the modified BN materials exhibit steeper slopes compared to pristine BN, indicating higher apparent kinetic constants. This effect is particularly evident for BN-OH under irradiation, where the highest slopes are observed, reflecting faster dye degradation. In contrast, pristine BN shows slopes close to zero, especially in the R6G system, highlighting its low photocatalytic activity. Similarly, in the PSO plots (C_0_/C vs. time), shown in Figure 7 for (a) R6G and (b) MB, BN-OH exhibits the highest linearity and apparent reaction rates, followed by BN-BM, while pristine BN shows a less defined behavior. These results suggest that surface modification not only increases the degradation rate but also promotes more efficient interactions between the material and dye molecules [37,61]. Additionally, the photocatalytic degradation data obtained under solar irradiation were further analyzed using the Behnajady–Modirshahla–Ghanbary (BMG) model, expressed as t/(1 − C/C_0_) = m + bt. This model is particularly suitable for heterogeneous photocatalytic systems, as it provides a simplified kinetic description that accounts for both the apparent initial degradation rate and the overall oxidation behavior of the catalyst. In this framework, parameter 1/m is associated with the apparent initial reaction rate, while parameter b is related to the overall oxidation behavior and reflects the tendency of the system to sustain the degradation process over time [62]. The corresponding linear fittings are shown in Figure 7e for R6G and Figure 7f for MB, where it can be observed that BN-BM and BN-OH samples exhibit an excellent correlation with the BMG model for both dyes. In contrast, pristine BN shows a poorer fit, indicating that its degradation behavior does not follow a well-defined photocatalytic pathway under the evaluated conditions. It is important to note that this model was applied exclusively to the experiments performed under solar irradiation, since it is more appropriate for describing photocatalytic processes involving reactive oxygen species rather than adsorption-dominated systems in dark conditions.

The kinetic constants and correlation coefficients (R^2^) obtained from both models are summarized in Table 4. In general, the pseudo-first-order (PFO) model provides a better fit for most systems under irradiation conditions, particularly in the case of methylene blue, where R^2^ values close to 0.99 are obtained for BN and above 0.94 for BN-BM. However, for the BN-OH material, a decrease in the PFO fitting quality is observed in some cases, which may be associated with the rapid kinetics of the process and the simultaneous contribution of multiple mechanisms (adsorption and photodegradation) [63]. For rhodamine 6G, the R^2^ values are generally lower compared to MB, especially for pristine BN. This behavior is attributed to the limited variation in dye concentration during the process, which reduces the sensitivity of the kinetic models and consequently the quality of the fit. In contrast, the modified materials (BN-BM and BN-OH) exhibit higher R^2^ values, confirming their enhanced efficiency and activity in dye degradation.

To complement this analysis, the photocatalytic data obtained under solar irradiation were also evaluated using the BMG model. As shown in Table 4, this model provides an excellent fit for the modified materials, particularly BN-OH, where R^2^ values approach unity for both dyes, and for BN-BM, which also shows a strong linear correlation. In contrast, pristine BN exhibits significantly lower R^2^ values, indicating that its degradation behavior does not follow a well-defined photocatalytic pathway under these conditions. Importantly, the applicability of the BMG model is directly related to the intrinsic two-stage nature of advanced oxidation processes, which involve an initial rapid degradation step followed by a slower reaction stage due to changes in reactive species availability and surface interactions [62]. This feature makes the BMG model particularly suitable for describing photocatalytic systems, where multiple reaction pathways and transient species coexist. From a physical perspective, the parameter 1/m shows a marked increase for the modified materials under irradiation. For methylene blue, BN-OH exhibits a significantly higher 1/m value compared to BN and BN-BM, indicating a much faster initial degradation stage. A similar trend is observed for rhodamine 6G, although with lower absolute values, reflecting the more complex degradation of this dye. This behavior is consistent with an enhanced generation of reactive oxygen species (ROS) during the early stages of the reaction, which has been associated with defect-rich surfaces and functionalized materials that promote catalytic activity [64]. Regarding the parameter b, the modified materials exhibit more consistent and moderate values compared to pristine BN. In the case of rhodamine 6G, the unusually high b value observed for BN indicates a non-ideal behavior, likely associated with limited degradation and the dominance of non-photocatalytic contributions. In contrast, BN-BM and BN-OH show values of b within a narrower range, suggesting a more stable and controlled degradation process. This can be interpreted as a more efficient transition between the fast initial stage and the slower regime, which is characteristic of systems governed by two-stage kinetics.

These results highlight that, while PFO and PSO models provide useful information about overall kinetics, they are limited to simplified single-mechanism descriptions. In contrast, the BMG model offers a more comprehensive representation of photocatalytic behavior under irradiation, as it captures both the initial reaction rate and the progressive evolution of the degradation process. In this context, the superior performance observed for BN-BM and BN-OH is associated with defect generation, increased surface area, and the incorporation of functional groups (–OH), which favor charge separation and the formation of reactive species [17], ultimately enabling a more efficient and sustained photocatalytic response.

### 2.7. Post-Reaction FTIR Analysis and Reactive Species Identification

**Figure 8:**
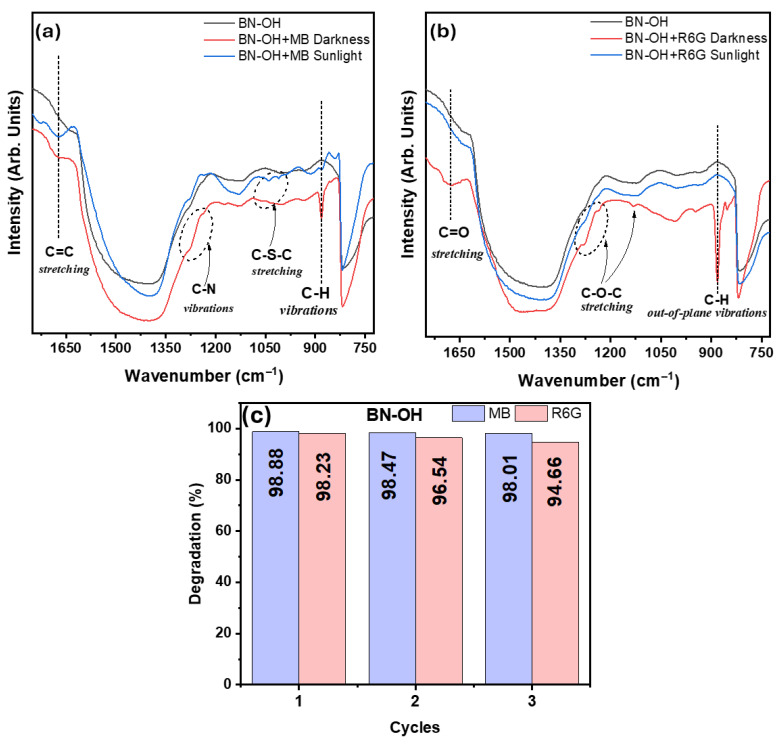
FTIR spectra of BN–OH before and after interaction with (**a**) MB and (**b**) R6G under dark and sunlight conditions and (**c**) Reusability performance of BN-OH for MB and R6G degradation over three consecutive cycles.

The FTIR spectra of BN, obtained after the adsorption experiments, revealed the presence of additional bands associated with the organic dye molecules (Figure 8). The analyses were performed on powder samples using diffuse reflectance spectroscopy with KBr in the mid-infrared region (500–4000 cm^−1^). For samples loaded with MB, characteristic bands were observed around 1600 cm^−1^ and 1200–1250 cm^−1^, corresponding to aromatic C=C stretching and C–N vibrations, respectively. Additionally, a contribution in the 1020–1050 cm^−1^ region was identified and assigned to C–S–C stretching vibrations, characteristic of the phenothiazine structure of the dye. Furthermore, a band around 885 cm^−1^ was observed, which is associated with C–H bending vibrations in the aromatic ring (out-of-plane) [65]. In contrast, systems containing R6G exhibited a distinctive band near 1650 cm^−1^ attributed to C=O stretching, along with signals in the 1500–1550 cm^−1^ and 1100–1200 cm^−1^ regions, associated with aromatic vibrations and C–O–C stretching of the xanthene structure [66]. Additionally, a band around 886 cm^−1^ was identified for both dyes, attributed to out-of-plane bending vibrations of aromatic C–H bonds, which was more pronounced in the R6G systems. The decrease in the intensity of these bands under solar irradiation suggests partial degradation of the dye molecules, confirming the photocatalytic activity of the materials [67].

The long-term stability and reusability of the BN–OH material were evaluated through consecutive photocatalytic cycles under identical experimental conditions, as shown in Figure 8c. The results demonstrate that BN–OH maintains a high degradation efficiency over four cycles for both dyes. In the case of methylene blue, the removal efficiency remains above 98% throughout all cycles, with only a negligible decrease from ~99% in the first cycle to ~98.01% in the fourth cycle. Similarly, for rhodamine 6G, the degradation efficiency shows a slight decline from ~99% to ~94.66% after four cycles. This minor reduction may be attributed to the partial accumulation of intermediate species on the catalyst surface or slight loss of active sites during repeated use. Nevertheless, the overall performance remains remarkably stable, indicating that BN–OH possesses good structural stability and reusability, which supports its potential application in wastewater treatment processes.

**Figure 9:**
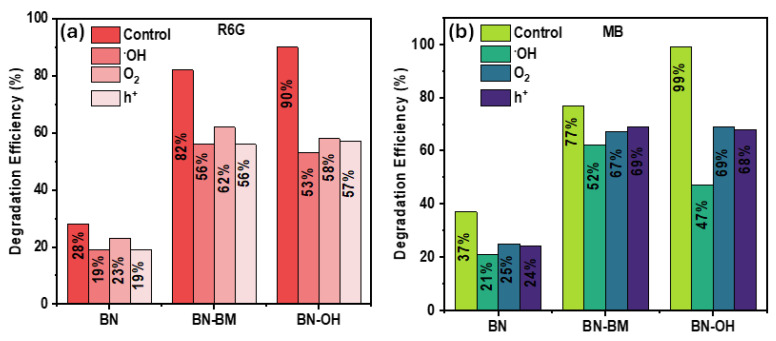
Scavenger tests for the photocatalytic degradation of (**a**) R6G and (**b**) MB over BN, BN-BM, and BN-OH under sunlight irradiation.

To identify the ROS responsible for the photocatalytic process, scavenger experiments were conducted using isopropanol (IPA, •OH), ascorbic acid (O_2_•^−^), and triethanolamine (TEOA, h^+^). The results are shown in Figure 9 for (a) MB and (b) R6G. For MB, the degradation efficiency significantly decreases in the presence of IPA, particularly for BN-OH (from 99% to 47%), indicating that •OH radicals are the dominant reactive species. The partial inhibition observed in the presence of O_2_•^−^ and h^+^ (69% and 68%, respectively) suggests a secondary synergistic contribution. For BN-BM, a similar but less pronounced trend is observed, while pristine BN shows low efficiency and minimal variation, confirming limited ROS generation. In contrast, for R6G, BN-OH exhibits a relatively uniform decrease in degradation efficiency (from 90% to 53–58%) in the presence of all scavengers, without a clearly dominant species, suggesting a multireactive mechanism. BN-BM shows comparable behavior (56–62%), whereas BN exhibits low activity (28%) with negligible influence of the scavengers.

## 3. Discussion

The obtained results demonstrate that mechanochemical treatment combined with NaOH-assisted hydroxylation effectively modifies the structural, surface, and electronic properties of h-BN, leading to enhanced adsorption and photocatalytic performance. These findings are consistent with previous reports indicating that defect engineering and surface functionalization play a key role in activating chemically inert layered materials for environmental applications.

Based on the morphological, structural, and spectroscopic evidence, it is proposed that the exfoliation and hydroxylation of h-BN during NaOH-assisted ball milling occur through a synergistic mechanochemical mechanism. Initially, impact and shear forces fragment the h-BN platelets and generate structural defects, particularly at edges and undercoordinated sites [39]. At these defect sites, boron atoms behave as electrophilic centers due to the intrinsic polarization of the B–N bond, making them susceptible to nucleophilic attack by OH^−^ ions in the alkaline medium. As a result, surface species such as B–OH and/or B–O^−^ are formed, while some nitrogen atoms may become protonated or remain in perturbed electronic environments, giving rise to N–H species or defective nitrogen sites. This process induces local rehybridization of boron centers from trigonal planar sp^2^ geometry toward distorted tetrahedral environments with partial sp^3^ character, without completely disrupting the basal lattice [40]. Furthermore, the incorporation of hydroxyl groups increases interlayer spacing, weakens van der Waals interactions, and enhances surface hydration, thereby facilitating progressive delamination and the formation of thinner structures [6,41]. These structural changes are typically associated with improved surface accessibility and may contribute to an increase in the effective surface area. Additionally, these modifications introduce defect states and promote charge carrier separation, which enhances light absorption and photocatalytic activity. In agreement with previous studies, defect-rich and hydroxyl-functionalized surfaces favor the generation of reactive oxygen species (ROS), which are essential for photocatalytic processes.

Overall, these results demonstrate that structural modifications of BN enhance ROS generation, with •OH radicals predominating in the degradation of methylene blue (MB), while a combined mechanism involving •OH, O_2_•^−^, and h^+^ is operative for rhodamine 6G (R6G) [18]. This behavior can be attributed to the increased defect density, surface functionalization with –OH groups, and improved charge carrier separation, all of which promote ROS formation [68]. From a mechanistic perspective, under irradiation, electrons in the conduction band react with dissolved O_2_ to generate O_2_•^−^, while holes in the valence band can either directly oxidize the pollutant or react with H_2_O/OH^−^ to produce •OH radicals [69]. The dominant role of •OH in MB degradation suggests a predominantly indirect oxidative pathway, whereas for R6G a more distributed mechanism involving both direct and indirect oxidation routes is observed [70,71]. The proposed photocatalytic degradation mechanism is detailed and summarized below, highlighting the generation and role of ROS during the process.

Photogenerated holes (h^+^) oxidize H_2_O/OH^−^ to produce hydroxyl radicals (•OH), while electrons (e^−^) reduce dissolved O_2_ to superoxide radicals (O_2_•^−^) [69].

hν → e^−^ + h^+^ (1)

h^+^ + H_2_O/OH^−^ → •OH (2)

e^−^ + O_2_ → O_2_•^−^ (3)

These primary species can undergo subsequent transformations, forming secondary oxidizing agents such as hydroperoxyl radicals (HO_2_•) and hydrogen peroxide (H_2_O_2_), which further contribute to •OH generation through cascade reactions [72,73].

O_2_•^−^ + H^+^ → HO_2_• (4)

2HO_2_• → H_2_O_2_ + O_2_ (5)

H_2_O_2_ + e^−^ → •OH + OH^−^ (6)

This sequence highlights the synergistic role of electrons and holes in sustaining ROS production, which is widely recognized as the main driving force in photocatalytic degradation processes [74].

Approaches based on electronic structure analysis and bond dissociation energy (BDE) have been widely used to elucidate the reactivity and degradation pathways of organic molecules under oxidative conditions, providing a theoretical framework to interpret photocatalytic processes [75]. In this context, the degradation pathway can be rationalized considering BDE principles, where specific bonds such as N–CH_3_ in methylene blue are more susceptible to cleavage under oxidative conditions. DFT studies have demonstrated that hydroxyl radicals preferentially attack these groups, leading to stepwise demethylation processes with favorable reaction energies [58]. In contrast, rhodamine 6G presents a more complex and rigid xanthene-based structure with a higher degree of conjugation, which enhances the stability of its molecular framework and results in a higher resistance to bond cleavage [76].

**Figure 10:**
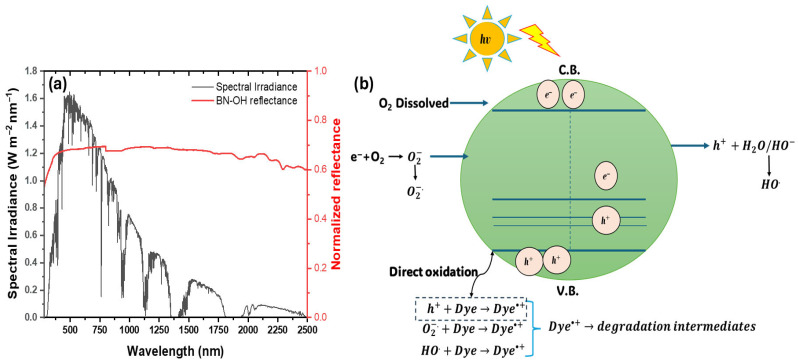
(**a**) Spectral overlap between the AM 1.5 solar irradiance spectrum and the normalized reflectance response of BN-OH; (**b**) proposed sunlight-driven photocatalytic mechanism involving defect-related surface states, charge carrier generation, ROS formation, and dye degradation. C.B. and V.B. denote the conduction band and valence band, respectively.

From another perspective, Figure 10a shows the superposition of the solar spectral irradiance, taken from the AM 1.5 ASTM G173 standard, and the normalized reflectance response of BN-OH. The solar irradiance exhibits a significant contribution in the UV–visible–NIR region, while BN-OH displays a broad optical response within this spectral range. BN-OH presents a sub-bandgap transition at around 2.24 eV, corresponding to approximately 554 nm, which falls within the visible region. This spectral overlap suggests that a fraction of the photons available under solar irradiation may interact with the BN-OH surface, particularly through defect-related states and oxygen-containing functional groups such as B–O/B–OH, as discussed in Section 2.3. Therefore, the optical response of BN-OH supports its sunlight-driven activity by correlating the availability of solar photons with the presence of active surface states capable of participating in photoinduced processes. Along with this, the proposed mechanism, shown in Figure 10b, is in good agreement with the experimental scavenger results and highlights the synergistic role of hydroxyl functionalization and defect generation in enhancing charge separation and ROS production. These findings position BN-OH as a promising material for advanced wastewater treatment applications.

Finally, future research should focus on optimizing mechanochemical parameters to control defect density and functional group distribution, as well as evaluating long-term stability and reusability under realistic conditions. Additionally, extending this strategy to other layered materials may provide new opportunities for the design of efficient photocatalysts.

## 4. Materials and Methods

Hexagonal boron nitride (h-BN) was purchased from ZYP Coatings Inc. (Oak Ridge, TN, USA), and sodium hydroxide (NaOH) from J.T. Baker (Radnor, PA, USA). The mechanically exfoliated sample (BN-BM) was prepared via a high-energy ball milling process. Briefly, 1 g of h-BN was placed in a milling reactor together with zirconia balls, maintaining a mass ratio of 1:10 (h-BN:balls). The system was processed in a high-energy ball mill for 3 h, yielding the material denoted as BN-BM. For surface hydroxylation, 10 mL of a 2 M NaOH solution were added to the BN-BM sample, followed by an additional milling step for 1 h. The resulting product was washed three times using a mixture of distilled water and ethanol (1:1 *v*/*v*) and centrifuged at 4000 rpm for 15 min in each cycle. The samples were then dried at 60 °C for 12 h. This washing and centrifugation procedure was applied to both BN-BM and the chemically treated sample, which was denoted as BN-OH. Finally, all samples were subjected to further characterization.

Phase identification was carried out by X-ray diffraction (XRD) using a Rigaku D/Max 2100 diffractometer (Rigaku Corporation, Akishima-shi, Tokyo, Japan) with CuKα radiation (λ = 1.5406 Å), operating at 40 kV and 30 mA, within a 2θ range of 10–90° and a step size of 0.02°. Morphological analysis was conducted by high-resolution scanning electron microscopy (H5R-SEM, JEOL 7610-F, JEOL Ltd., Akishima, Tokyo, Japan) at 2 kV with a working distance of 6.0 mm. Thermogravimetric analysis (TGA) was performed using a Perkin Elmer TGA 4000 analyzer (PerkinElmer, Inc., Waltham, MA, USA). The experiments were carried out at a heating rate of 10 °C/min from 30 °C to 600 °C. XPS spectra were collected using an X-ray photoelectron spectrometer PHI 5100 (Physical Electronics, Inc., Chanhassen, MN, USA). High-resolution XPS (HR-XPS) spectra were acquired with a pass energy of 15 eV and a step size of 0.05 eV. Elemental composition was determined by energy-dispersive X-ray spectroscopy (EDS). Fourier transform infrared spectroscopy (FTIR) in diffuse reflectance mode (DRIFTS) was performed using a Perkin Elmer Spectrum GX spectrometer (PerkinElmer, Inc., Waltham, MA, USA) with 24 scans and a resolution of 4 cm^−1^. Adsorption experiments were carried out using 10 mg of boron nitride in aqueous solutions of methylene blue (MB, C_16_H_18_ClN_3_S) and rhodamine 6G (R6G, C_24_H_32_N_2_O_3_), with initial concentrations ranging from 3 to 68 mg/L. The suspensions were stirred for 24 h prior to UV–Vis analysis using an Ocean Optics spectrophotometer (Ocean Optics, Orlando, FL, USA). The experimental data were fitted to Freundlich, Langmuir, and Temkin models. Photodegradation tests were performed using aqueous dye solutions (1.44 × 10^−4^ M). A total of 0.050 g of bulk h-BN, BN-BM, and BN-OH was added to 50 mL of solution under static conditions. Experiments were conducted under dark conditions and direct solar irradiation (20°43′48.1″ N, 100°28′11.1″ W; 1820 m a.s.l.). All solar irradiation tests were carried out between 11:00 and 14:00 h, corresponding to the time window in which the solar intensity remained within the range of 930–980 W m^−2^. The experiments were performed exclusively under clear sky conditions, with an ambient temperature between 30 °C and 33 °C. Additionally, reusability tests were conducted through three consecutive cycles using the BN-OH sample under identical experimental conditions.

## 5. Conclusions

In this study, a synergistic mechanochemical strategy combining ball milling and NaOH-assisted treatment was successfully employed to induce partial hydroxylation and exfoliation of hexagonal boron nitride (h-BN), yielding materials with enhanced structural, surface, and electronic properties. The structural and spectroscopic analyses (XRD, FTIR, XPS, and TGA) confirmed that the applied treatments progressively increase defect density, oxygen-containing functional groups, and interlayer spacing, while reducing crystallinity from 80.9% (BN) to 53.5% (BN-BM) and 39.1% (BN-OH), as well as decreasing crystallite size from 20.3 nm to 13.3 nm and 10.8 nm, respectively. In parallel, band gap narrowing from 5.2 eV (BN) to 5.08 eV (BN-BM) and 4.87 eV (BN-OH), along with the appearance of defect-related sub-bandgap states, evidences the progressive electronic modulation induced by mechanochemical treatment. Importantly, the inclusion of BN-BM as an intermediate material allowed isolating the specific contribution of the ball milling process. BN-BM exhibited a significant enhancement in performance compared to pristine BN, reaching adsorption capacities of ~147 mg/g for MB and degradation efficiencies up to 97.8% (MB) and 94.7% (R6G) under solar irradiation. This improvement is attributed to defect generation, partial exfoliation, and increased surface reactivity induced by ball milling. These results demonstrate that mechanochemical activation alone is sufficient to substantially improve both adsorption and photocatalytic activity, establishing BN-BM as a critical intermediate stage in the modification pathway. In particular, the formation of B–OH and B–O species in BN-OH played a critical role in further modifying surface polarity and promoting charge transfer processes. These modifications translated into improved dispersion behavior, higher negative surface charge, and enhanced interaction with aqueous media. From a performance standpoint, BN-OH exhibited superior adsorption and photocatalytic activity compared to BN and BN-BM, achieving adsorption capacities up to 248 mg/g for MB and near-complete degradation (~99%) for both MB and R6G. The adsorption studies revealed that the process is governed by a heterogeneous mechanism involving electrostatic interactions, π–π stacking, hydrogen bonding, and defect-mediated adsorption. MB showed higher adsorption capacity than R6G, mainly due to its smaller and more planar molecular structure, which facilitates access to active sites. Photocatalytic results demonstrated that surface modification significantly enhances dye degradation under solar irradiation, with a progressive improvement from BN to BN-BM and BN-OH. The kinetic analysis indicated that the degradation process is predominantly described by pseudo-first-order behavior, with BN-BM already showing a marked increase in apparent rate constants compared to pristine BN, confirming the key role of defect engineering. Scavenger experiments revealed that hydroxyl radicals (•OH) are the dominant reactive species in MB degradation, whereas a combined contribution of •OH, O_2_•^−^, and h^+^ governs the degradation of R6G. This difference highlights the influence of molecular structure on the degradation pathway and confirms that defect engineering and hydroxyl functionalization enhance ROS generation and charge carrier separation. Overall, this work demonstrates that the controlled hydroxylation of exfoliated h-BN is an effective strategy to transform an intrinsically insulating material into a multifunctional system with significant adsorption and photocatalytic capabilities. Importantly, the results highlight that ball milling is not merely a preparative step, but a key mechanochemical activation process that enables defect formation and significantly enhances performance even prior to chemical functionalization. The developed BN-OH material shows strong potential for application in water treatment processes, particularly for the removal of persistent organic dyes under environmentally relevant conditions. From a strategic perspective, these findings position hydroxylated boron nitride as a competitive and sustainable alternative within the field of advanced oxidation processes, opening opportunities for its integration into scalable environmental remediation technologies.

## Figures and Tables

**Table 1:** XRD-derived structural parameters of h-BN, BN-BM, and BN-OH.

Sample	2θ (002)	d_002_ (Nm)	FWHM (°)	Crystallite Size (nm)	Crystallinity (%)
h-BN	26.75	0.332	0.402	20.3	80.9
BN-BM	26.68	0.333	0.612	13.3	53.5
BN-OH	26.58	0.335	0.758	10.8	39.1

**Table 2:** Isotherm parameters for MB and R6G adsorption on BN-based materials.

Material	Dye	Langmuir			Freundlich			Temkin		
		*q_e_* (Mg/g)	*b* (L/Mg)	*R* ^2^	*K*	*n*	*R* ^2^	*A* (L/g)	*B* (J/Mol)	*R* ^2^
BN	MB	24.43 ± 2.13	1.086 ± 0.02	0.9979	23.57 ± 1.5	2.43	0.9627	0.2066 ± 0.0231	128.61 ± 1.81	0.9919
BN-BM		74.97 ± 3.20	0.07826 ± 0.01462	0.9271	18.95 ± 0.49	0.45	0.9971	1.2207 ± 0.101	14.20 ± 0.154	0.9810
BN-OH		248.61 ± 35.19	0.1743 ± 0.0177	0.8769	55.22 ± 6.68	0.4258	0.9568	5.535 ± 0.0317	38.58 ± 5.94	0.8578
BN	R6G	112.86 ± 0.90	0.1079 ± 0.0027	0.9992	23.57 ± 2.49	0.3724	0.9627	1.086 ± 0.0547	24.43 ± 0.422	0.9979
BN-BM		147.96 ± 12.67	0.1139 ± 0.0295	0.9264	31.11 ± 2.41	0.380	0.9807	2.379 ± 0.9270	25.83 ± 2.9134	0.9182
BN-OH		215.37 ± 28.99	0.2828 ± 0.1029	0.8720	58.56 ± 4.21	0.4033	0.9698	7.519 ± 3.392	34.01 ± 4.96	0.8705

**Table 3:** Comparative performance of reported photocatalysts for dye degradation under different irradiation conditions, including degradation efficiency and reaction time.

Materials	Dye	Light Source	Time (Min)	Degradation (%)	Reference
TiO_2_/GO	MB	Visible	140 min	97.5%	[48]
MoS_2_/g-C_3_N_4_/TiO_2_	MG	Visible	60 min	85%	[49]
GO/CeO_2_	MB	UV	80 min	94.2%	[50]
ZIF-8	MB	UV	120 min	82.3%	[51]
ZnO/Ag	R6G	UV	120 min	90%	[52]
TiO_2_/SiO_2_	RhB	Visible	210 min	100%	[53]
AgNps (green synthesis)	RhB	UV	90 min	93.56	[54]
BN/Fe_2_O_3_	MB	Visible	180 min	91%	[55]
BN-OH	MB/RhB/MO	Visible	90 min	MB: 12.3% RhB: 45.2% MO: 7.4%	[18]
Zn/BNQDs	MB/MO	UV	50 min	MB: 99.9% MO: 97.9%	[56]
BN-OH	MB/R6G	Solar	60 min	MB: 98.6% R6G: 90.6%	This work

**Table 4:** Kinetic parameters of pseudo-first-order (PFO), pseudo-second-order (PSO) and Behnajady–Modirshahla–Ghanbary (BMG) models for MB and R6G degradation by BN, BN-BM, and BN-OH under dark and sunlight conditions.

			PFO		PSO		BMG		
Dye	Sample	Conditions	k (min^−1^)	R^2^	k′ (min^−1^)	R^2^	1/m (min^−1^)	b	R^2^
	BN	Dark	0.0023	0.804	0.0028	0.843	-	-	-
		Sunlight	0.0048	0.988	0.0066	0.978	0.0063	1.0295	0.619
Methylene blue	BN-BM	Dark	0.0166	0.925	0.0594	0.896	-	-	-
		Sunlight	0.0329	0.942	0.3723	0.899	0.0638	0.8752	0.995
	BN-OH	Dark	0.0176	0.898	0.0788	0.950	-	-	-
		Sunlight	0.0281	0.570	0.6878	0.871	1.626	1.0062	0.999
Rhodamine 6G	BN	Dark	0.0012	0.587	0.0014	0.606	-	-	-
		Sunlight	0.0002	0.611	0.0003	0.615	0.0024	20.592	0.849
	BN-BM	Dark	0.0120	0.955	0.0297	0.960	-	-	-
		Sunlight	0.0248	0.961	0.1387	0.938	0.0303	0.7554	0.967
	BN-OH	Dark	0.0121	0.929	0.0314	0.956	-	-	-
		Sunlight	0.0356	0.962	0.8515	0.717	0.1404	0.9543	0.999

## Data Availability

The data supporting the findings of this study are included within the article. Additional data are available from the corresponding author upon reasonable request.
